# *Lycium ruthenicum* Murr. Polysaccharide Attenuated Inflammatory Response and Intestinal Flora Dysbiosis in LPS-Induced Acute Lung Injury in Mice

**DOI:** 10.3390/nu17182944

**Published:** 2025-09-12

**Authors:** Naiyan Lu, Shuhua Xu, Wen Xiang, Xue Mei, Hanwen Hu, Xue Tang, Xuelei Gong, Xun Wang

**Affiliations:** 1 School of Food Science and Technology, Jiangnan University, Wuxi 214126, China; lunaiyan@jiangnan.edu.cn (N.L.); 6230112100@stu.jiangnan.edu.cn (S.X.); 6230112060@stu.jiangnan.edu.cn (X.M.); 6240112130@stu.jiangnan.edu.cn (H.H.); tangxue@jiangnan.edu.cn (X.T.); 2 School of Medicine, Jiangnan University, Wuxi 214122, China; 1120200668@mail.nankai.edu.cn; 3 School of Medicine, Nankai University, Tianjin 300350, China; 4 Medical School, Nantong University, Nantong 226007, China; 2331320036@stmail.ntu.edu.cn

**Keywords:** *Lycium ruthenicum* Murr. polysaccharide, lung injury, inflammation, intestinal microflora

## Abstract

**Background/Objectives:** Acute lung injury (ALI) represents a life-threatening respiratory syndrome characterized by dysregulated pulmonary inflammation, alveolar-capillary barrier dysfunction, and gut-lung axis impairment. Although *Lycium ruthenicum* polysaccharide (LRP) possesses documented anti-inflammatory properties, its role in ALI remains systematically unexplored. This study aimed to investigate the protective effects of LRP against lipopolysaccharide (LPS)-induced ALI. **Methods:** In vitro, A549 cells were subjected to injury induction with 10 μg/mL LPS. In vivo, male C57BL/6J mice were randomly allocated to four groups and, respectively, administered 100 mg/kg LRP, 400 mg/kg LRP, or normal saline for 7 days prior to ALI induction via intratracheal LPS instillation (5 mg/kg). **Results:** LRP restored viability in LPS-injured A549 cells and attenuated their inflammatory responses. Histopathological analysis demonstrated that high-dose LRP (H-LRP) significantly reduced alveolar collapse and inhibited inflammatory cell infiltration in bronchoalveolar lavage fluid (BALF) compared to the LPS group. The H-LRP group exhibited marked downregulation of pro-inflammatory cytokines (TNF-α, IL-1β, IL-6) concomitant with upregulation of the anti-inflammatory cytokine IL-10. Intestinal microbiome sequencing confirmed LRP-mediated restoration of gut microbiota homeostasis, evidenced by a 2.2-fold increase in commensal Bacteroides and decreased abundance of pathogenic Escherichia-Shigella. **Conclusions:** These findings establish LRP as a protective agent against ALI and suggest its potential utility as an adjuvant therapeutic candidate for enhanced pulmonary protection.

## 1. Introduction

Acute lung injury (ALI), a critical syndrome often progressing to acute respiratory distress syndrome (ARDS), is characterized by uncontrolled pulmonary inflammation, alveolar-capillary barrier dysfunction, and non-cardiogenic pulmonary edema [1]. Its high mortality and long-term morbidity stem from dysregulated inflammation [2,3,4]. Pathogen-associated molecular patterns, such as lipopolysaccharide (LPS), activate macrophages to release pro-inflammatory cytokines like TNF-α, IL-6, and IL-1β [5,6]. This cytokine release recruits neutrophils into the lung tissue, leading to lipid peroxidation, mitochondrial dysfunction, and endothelial/epithelial cell apoptosis [7]. Gut dysbiosis exacerbates ALI via the “gut-lung axis” [8]—intestinal barrier compromise enables endotoxin translocation, while reduced probiotic-derived short-chain fatty acids (SCFAs) diminish inhibition of pulmonary neutrophil activation [9,10,11].

Currently, the clinical management of ALI relies on medications such as glucocorticoids. However, these therapeutic strategies face limitations like limited efficacy and significant side effects, which restrict their application in ALI treatment. Long-term use of anti-inflammatory drugs (e.g., dexamethasone) can induce immunosuppression, gut microbiota disruption, and secondary infections. More critically, single-target therapeutic interventions are insufficient to concurrently block the complex, cascading pathological network involving the interaction between inflammatory pathways and the gut-lung axis [12]. Plant polysaccharides, natural polymers with multi-target bioactivity and high biosafety [13], offer advantages by suppressing TLR/NF-κB pathways [14,15] and enhancing epithelial barrier integrity [16,17]. Consequently, plant polysaccharides can exert a synergistic therapeutic effect characterized as “anti-inflammatory and gut microbiota regulation” through suppressing inflammatory signaling pathways and restoring gut microbiota homeostasis. This provides a novel strategy for overcoming the limitations in current ALI therapies [18,19].

*Lycium ruthenicum* Murr. Polysaccharide (LRP) is the primary bioactive constituent of the plateau-dwelling medicinal plant *Lycium ruthenicum*, which has been shown to alleviate inflammatory responses and modulate the gut microbiota [20,21]. Moreover, aqueous extracts of *Lycium ruthenicum* have been demonstrated to mitigate neuroinflammation and regulate the gut microbial community [22]. LRP shows distinctive therapeutic value in inflammatory diseases. Its sulfated structure and complex monosaccharide composition confer potent anti-inflammatory activity [23]—exemplified by suppression of pro-inflammatory cytokine release through blockade of the TLR4/NF-κB signaling pathway [24]. Furthermore, LRP exhibits potential gut microbiota-modulating functions, acting as a prebiotic to promote proliferation of Lactobacillus and Bifidobacterium while inhibiting pathogenic Enterobacteriaceae colonization [25]. Although many studies have investigated the effects of LRP on metabolic syndrome and hepatic injury, its therapeutic potential for ALI remains unexplored [26,27,28].

However, the role of LRP in ALI to attenuate the inflammatory response and modulate the intestinal flora remains unclear. Therefore, this study aims to investigate the protective effects of LRP against LPS-induced ALI. We extracted and characterized LRP, then established in vitro and in vivo ALI models via LPS. The pulmonary protective mechanisms of LRP were probed by assessing inflammatory markers in lung tissues and conducting gut microbiota sequencing. Our findings will establish a theoretical and experimental foundation for developing LRP as a natural therapeutic agent for ALI, thereby expanding the applicability of the gut-lung axis theory in respiratory diseases.

This study demonstrates for the first time LRP’s protection against LPS-induced ALI through multidimensional anti-inflammatory-microbiota regulatory mechanisms. We extracted LRP, established in vitro/vivo ALI models, assessed pulmonary inflammation biomarkers, and analyzed gut microbiota. These findings establish LRP as a natural ALI therapeutic candidate and expand the gut-lung axis framework in respiratory diseases, revealing significant scientific and translational potential.

## 2. Materials and Methods

### 2.1. Materials and Reagents

*Lycium ruthenicum* Murr. were purchased from Qinghai Zaokang Goji Berries Co., Ltd. (Zhongwei, China). The supplier of mannose, rhamnose, galacturonic acid, glucose, galactose, and arabinose was Macklin Biochemical Technology Co. (Shanghai, China). LPS was provided by Sigma Aldrich (Saint Louis, MO, USA) for reagent use. Calcein-AM/PI Cell Viability Assay Kit was purchased from Biosharp Biotechnology Co. CCK-8 Assay Kit and H&E Staining Kit were purchased from Beijing Solarbio Science & Technology Co., Ltd. (Beijing, China) BCA Kit and 4% paraformaldehyde fixative were purchased from Shanghai Biyuntian Biotechnology Co. (Shanghai, China). Mouse Interleukin 6 (IL-6) Kit, Mouse Interleukin 10 (IL-10) Kit, Mouse Interleukin 1β (IL-1β) Kit and Mouse Tumour Necrosis Factor (TNF-α) Kit were purchased from Xiamen Huijia Biotechnology Co. (Xiamen, China). All other chemical reagents were of analytical grade and purchased from Sinopharm Chemical Reagent (Shanghai, China).

### 2.2. Preparation of LRP

LRP was extracted according to the method of Qin, with slight modifications [29]. Briefly, the dried LRM was crushed to obtain LRM powder. A certain amount of the powder was taken and mixed with ultrapure water at a material-liquid ratio of 1:10 (g/mL) and extracted by ultrasonic extraction at 100 °C for 60 min. The resulting extract was centrifuged at 8000 r/min for 15 min, and the filtrate was collected. This step was repeated, and the mixture was concentrated to one-third of its original volume by rotary evaporation at 50°C. Afterwards, the filtrate was collected by deproteinisation using the trichloroacetic acid method. The filtrate was precipitated with 4-fold volume of anhydrous ethanol at 4 °C overnight. The precipitate was then resuspended and lyophilized to obtain LRP.

### 2.3. Characterization of LRP

5 mg of LRP were mixed with 500 mg of dry KBr powder, ground, pressed into tablets, and measured in the wave number range of 4000–400 cm^−1^ using Fourier transform infrared spectroscopy (FTIR, Thermo Fisher Scientific, Waltham, MA, USA). High-performance gel permeation chromatography (HPGPC, Wyatt Technology, Santa Barbara, CA, USA) was used to measure polysaccharide molecular weights. The ultraviolet-visible (UV-vis) spectral analysis of LRP was conducted in the UV range with a spectral range between 190 and 400 nm.

After being hydrolyzed for two hours at 110 °C with trifluoroacetic acid, the LRP was blown dry with nitrogen, redissolved in ultrapure water, and filtered through 0.22 µm filters. The monosaccharide composition of LRP was determined by high-pressure ion chromatography (HPIC, Thermo Fisher Scientific, USA).

### 2.4. Cell Culture

The lung epithelial cell line A549 was obtained from Procell Life Science & Technology Co., Ltd. (Wuhan, China). A549 cells were cultured in Kaighn’s modified Ham’s F-12K medium containing 10% fetal bovine serum and 1% Penicillin–Streptomycin Solution at 37 °C with 5% CO_2_. Cell passaging was performed when the cells reached 80–90% confluency. The third-passage cells were used for subsequent experiments.

### 2.5. CCK-8 Assay

To investigate the effects of LRP on LPS-induced A549 cells, the cells were cultured in 96-well plates and divided into six groups. Sterile LRP solutions (filtered through a 0.22 μm membrane) were diluted in serum-free DMEM/F12 medium. Group 1 received DMEM/F-12 basal medium for 48 h, while Group 2 was pre-treated with basal medium for 24 h, followed by 10 µg/mL LPS for an additional 24 h. Groups 3–6 were administered LRP at increasing concentrations (50 μg/mL, 100 μg/mL, 150 μg/mL, 200 μg/mL) for 24 h before LPS stimulation. Cell viability was measured using the CCK-8 kit.

### 2.6. Living/Dead Cell Staining

A549 cells were seeded into 24-well plates for viability assessment via the Calcein-AM/PI dual-staining kit. After grouping, cells were stained with Calcein-AM (green fluorescence for live cells) and PI (red fluorescence for dead cells), washed with PBS, and imaged under a fluorescence microscope. Fluorescence intensity ratios were analyzed to calculate survival rates.

### 2.7. Animals and Experimental Protocol

Forty-eight male C57BL/6J mice (8 weeks old, body weight 20–25 g) were housed under standard environmental conditions with free access to a standard diet and ultrapure water. All animal experiments were approved by the Animal Ethics Committee of Jiangnan University and conducted in strict compliance with animal welfare guidelines (JN.No20241230c0600227[686]).

After 1 week of acclimatization, all mice were stratified by body weight and randomly assigned into four groups (*n* = 12) using a computer-generated random number table: Control group, LPS group, low dose LRP group (L-LRP), and high dose LRP group (H-LRP). The L-LRP and H-LRP groups were orally administered 100 mg/kg and 400 mg/kg LRP aqueous solution daily [30,31], respectively, while the Control and LPS groups received equivalent volumes of saline for 7 consecutive days. One hour after the final administration on day 7, mice in the LPS, L-LRP, and H-LRP groups underwent intratracheal instillation of LPS (5 mg/kg) under pentobarbital anesthesia, whereas the Control group received equivalent saline.

Twenty-four hours post-LPS challenge, mice were anesthetized, blood was collected via orbital bleeding, and euthanasia was performed by cervical dislocation. Blood samples were left at room temperature for 1 h before centrifugation to yield serum, which was stored at −80 °C. In each group, 6 mice underwent bilateral bronchoalveolar lavage for inflammatory cell count and cytokine analysis. The remaining 6 mice were used for lung wet/dry weight ratio (W/D) determination (left lung). The right lower lung lobe was fixed in 4% paraformaldehyde for histopathology, while residual lung tissue was flash-frozen in liquid nitrogen and stored at −80 °C for biochemical assays.

### 2.8. Hematoxylin & Eosin (H&E) Staining

The lung tissues were embedded in paraffin and sectioned into 6 μm slices using a microtome. The slices were floated in a water bath to flatten the wax, followed by deparaffinization with graded concentrations of xylene, ethanol, and distilled water. Finally, hematoxylin and eosin (H&E) staining was performed according to the manufacturer’s instructions.

### 2.9. Collection of BALF and Total Cells Count

Each mouse underwent three alveolar lavages with 1 mL of PBS to collect bronchoalveolar lavage fluid (BALF). After allowing the BALF to settle and stratify, it was centrifuged at 4 °C and 3000× *g* for 10 min, and the resulting cell pellet was resuspended in PBS for inflammatory cell counting. The total protein concentration in BALF was measured using a BCA protein assay kit, and cytokine levels in BALF were quantified.

### 2.10. Evaluation of the Lung Wet/Dry Weight Ratio

After euthanizing the mouse by cervical dislocation, the right lung is excised and weighed to determine its “wet weight.” The right lung is then dried in an oven at 80 °C for 72 h and weighed again to determine its “dry weight.” The wet/dry (W/D) weight ratio of the lung is a standard measure of pulmonary edema.

### 2.11. Measurement of Cytokine Levels in BALF and Serum

The levels of interleukin-1β (IL-1β), interleukin-6 (IL-6), tumor necrosis factor-α (TNF-α), and interleukin-10 (IL-10) in mouse serum and cell supernatants were measured according to the manufacturer’s instructions of the respective enzyme-linked immunosorbent assay (ELISA) kits.

### 2.12. 16S rRNA Analysis

The amplification and purification process targeted the V3-V4 region of the bacterial 16S rRNA gene. Fresh cecal contents were sent to Ling Gene Biotech Ltd. (Shanghai, China) for microbiome analysis. The amplification and purification focused on the V3-V4 region of the bacterial 16S rRNA gene, and the products were quantified and homogenized for Illumina PE250 library construction and sequencing. Subsequently, operational classification unit (OTU) clustering was performed (with confidence intervals above 97%).

### 2.13. Statistical Analysis

The results of cell and animal experiments were statistically analyzed using GraphPad 9.0, and all data were shown as mean ± standard deviation (Mean ± SD). Significant differences in the analyzed data were compared using one-way ANOVA followed by Tukey’s multiple comparison test (* *p* < 0.05, ** *p* < 0.01, *** *p* < 0.001, **** *p* < 0.0001, ns indicates no significant difference).

## 3. Results

### 3.1. Extraction and Characterization of LRP

**Figure 1:**
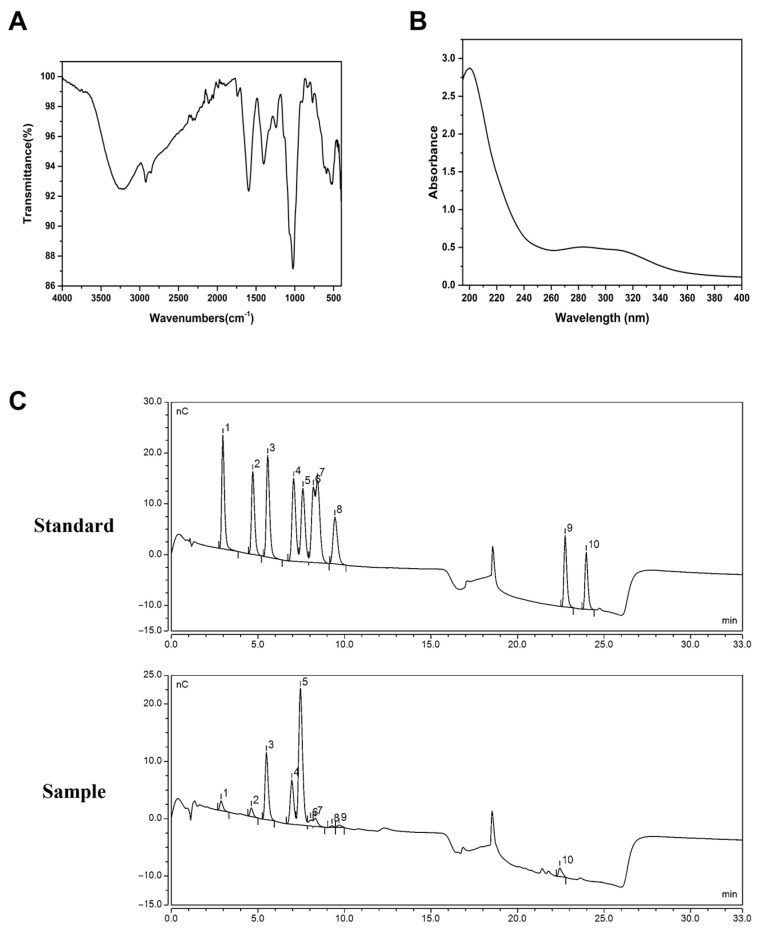
Characterization of LRP. (**A**) Characteristic FT-IR spectrum of LRP. (**B**) UV-Vis spectrum of LRP in the range of 190–400 nm. (**C**) The HPLC profiles of monosaccharide standards and LRP. 1. Fucose; 2. Rhamnose; 3. Arabinose; 4. Galactose; 5. Glucose; 6. Xylose; 7. Mannose; 8. Fructose; 9. Galacturonic acid; 10. Glucuronic acid. Abbreviations: FT-IR, Fourier Transform Infrared Spectroscopy; HPLC, High-Pressure Ion Chromatography.

LRP was isolated using the hot water extraction method, and both its yield and chemical composition were determined. The structure of LRP was characterized using UV-Vis and FT-IR spectroscopy. In the Fourier Transform Infrared (FT-IR) spectrum of LRP (Figure 1A), the absorption peak observed at 1055 cm^−1^ indicated the presence of the pyranose form, suggesting the existence of pyranose glycosidic bonds, while the peak at 871 cm^−1^ demonstrated the β-configuration of these glycosidic bonds. A weak but distinct peak was seen at 1736 cm^−1^, characteristic of the C=O stretching vibration of glucuronic acid. The broad absorption band detected at 3392 cm^−1^ was due to the O-H stretching vibration. Collectively, these peaks were characteristic of polysaccharides, confirming the polysaccharide nature of LRP [32]. Additionally, a weak peak detected at 818 cm^−1^ was consistent with arabinose-furan ring vibration, supporting the presence of arabinose in the monosaccharide composition. In the UV-Vis spectrum (Figure 1B), no characteristic absorption bands for proteins or nucleic acids were observed in the 260–280 nm range, confirming the absence of these contaminants in the purified LRP [33]. Subsequently, the molecular weight and monosaccharide compositions of the LRP fractions were analyzed by high-performance liquid chromatography (HPLC), resulting in a molecular weight of 59,432 Da. As shown in Figure 1C, the results indicated that LRP was a heteropolysaccharide primarily composed of D-glucose (50.02%), L-arabinose (20.45%), and D-galactose (14.6%).

### 3.2. LRP Restored Cell Viability and Alleviated Inflammatory Response of LPS-Injured A549 Cells

**Figure 2:**
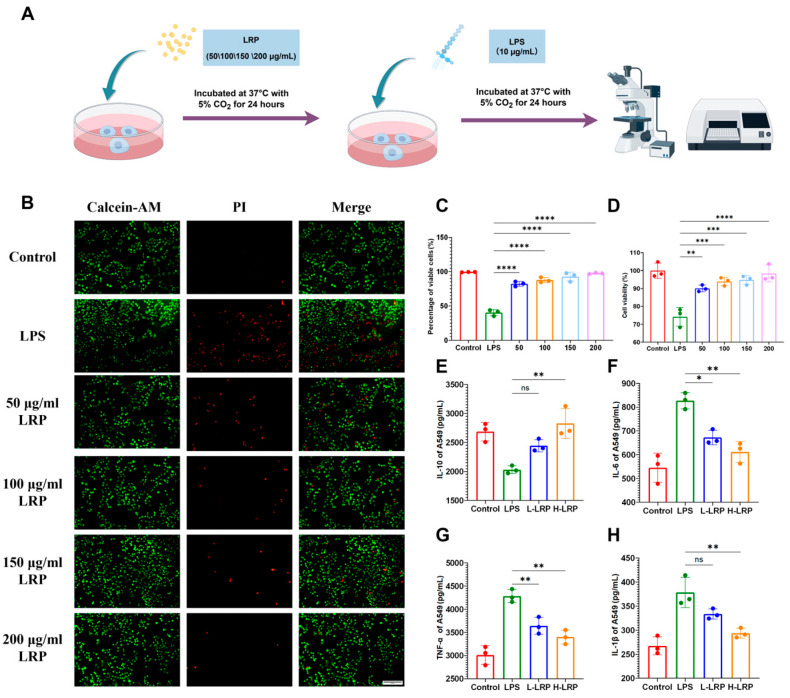
LRP restored cell viability and alleviated the inflammatory response of LPS-injured A549 cells. (**A**) Cell Experiment Flowchart. (**B**,**C**) Fluorescent images of live/dead cell staining experiments on LPS-injured A549 cells (×200, scale bar: 100 μm). The live cells were stained green while the dead cells were stained red. (**D**) Results of CCK-8 experiments on LPS-injured A549 cells. (**E**–**H**) Effects of LRP on inflammatory cytokines TNF-α, IL-6, IL-1β, and IL-10 in LPS-stimulated A549 cells. Data are presented as mean ± SD. Abbreviations: CCK-8, Cell Counting Kit-8; TNF-α, Tumor Necrosis Factor-alpha; IL-6, Interleukin-6; IL-1β, Interleukin-1 beta; IL-10, Interleukin-10. * *p* < 0.05, ** *p* < 0.01, *** *p* < 0.001, **** *p* < 0.0001, ns indicates no significant difference.

Figure 2B–D illustrates the effect of LRP on the viability of LPS-induced A549 cells. For LPS-injured A549 cells, all concentrations of LRP exhibited a partial protective effect, with the 200 μg/mL concentration of LRP showing the optimal protection. Next, we investigated the anti-inflammatory properties of LRP in vitro. The results revealed that LRP administration reduced levels of IL-1β, IL-6, and TNF-α in LPS-induced A549 cells while upregulating IL-10 expression (Figure 2E–H).

### 3.3. LRP Ameliorated Lung Histopathology and Pulmonary Oedema in ALI Mice

**Figure 3:**
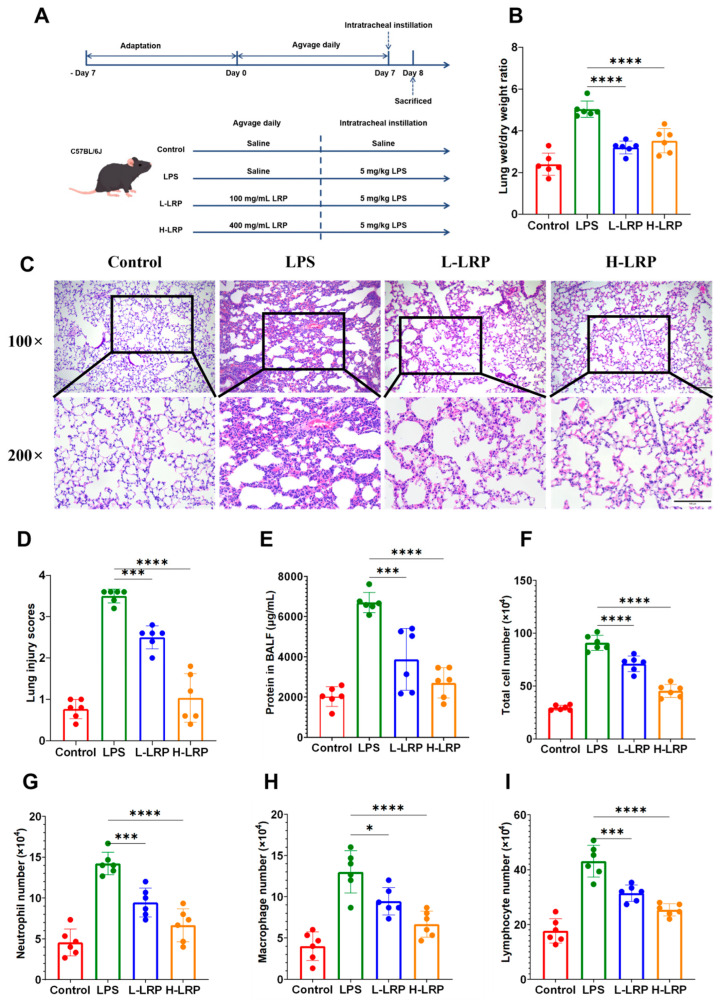
LRP attenuated LPS-induced acute lung injury. (**A**) Animal experiment flow chart. (**B**) Effect of LRP on the lung wet/dry ratio. (**C**,**D**) Results of H&E staining revealed the recovery effect of LRP on lung tissues (×200, scale bar: 100 μm). (**E**) Results of total protein levels in BALF. (**F**–**I**) Inflammatory cell counts in the BALF of mice with LPS-induced ALI. Abbreviations: H&E staining, Hematoxylin and Eosin staining; BALF, Bronchoalveolar Lavage Fluid. Data are presented as mean ± SD. * *p* < 0.05, *** *p* < 0.001, **** *p* < 0.0001.

As illustrated in Figure 3C, LPS induced significant structural damage to alveoli accompanied by marked inflammatory infiltration. The lung injury score in the LPS group was significantly higher than that in the control group. However, pretreatment with LRP via oral administration markedly attenuated alveolar wall thickening, prevented alveolar collapse, and minimized hemorrhage in lung tissues. At the same time, LPS exposure led to a significant increase in both lung W/D weight and total protein levels, indicative of pulmonary edema. In contrast, prophylactic LRP administration effectively mitigated lung edema in ALI mice. Subsequently, inflammatory cell counts were performed at 24 h post-LPS injection. Compared to the LPS group, LRP pretreatment resulted in significant reductions in total cell counts, including macrophages, neutrophils, and lymphocytes (Figure 3F–I).

### 3.4. LRP Alleviated Inflammatory Responses in ALI Mice

**Figure 4:**
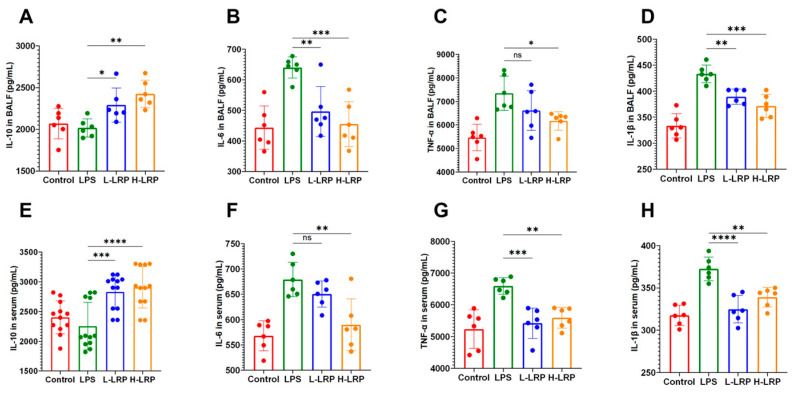
LRP modulated inflammatory cytokine levels in BALF and serum. (**A**–**D**) The levels of inflammatory marker variations in BALF. (**E**–**H**) The levels of inflammatory marker variations in serum. Data are presented as mean ± SD. * *p* < 0.05, ** *p* < 0.01, *** *p* < 0.001, **** *p* < 0.0001, ns indicates no significant difference.

To further investigate the effects of ALP on LPS-induced pulmonary inflammation, ELISA kits were used to measure the levels of inflammatory mediators in BALF (bronchoalveolar lavage fluid) and serum of mice. As shown in Figure 4, LRP administration significantly enhanced the expression of the anti-inflammatory cytokine IL-10 while suppressing the release of pro-inflammatory cytokines (IL-1β, IL-6, and TNF-α) in both BALF and serum.

### 3.5. LRP Regulated the Intestinal Microbiota in ALI Mice

**Figure 5:**
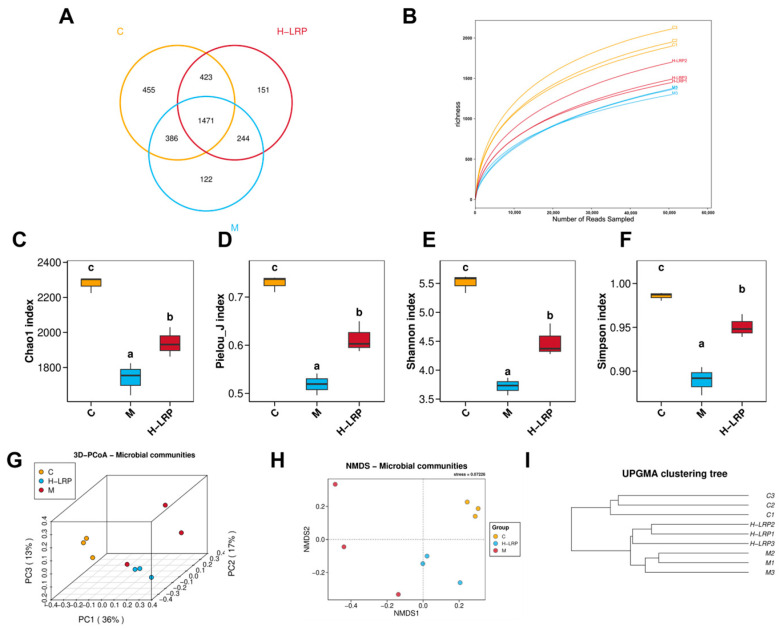
LRP improved the composition of gut microbes in mice with ALI. (**A**) OUT dilution profile of gut flora. (**B**) Wayne plots identifying OTUs that are common and endemic across groups of samples. (**C**–**F**) Intestinal microflora α-diversity analyses (Chao1 index, Pielou-J index, Shannon index, and Simpson index) assay (*n* = 3). (**G**) 3D-PCoA plot of the intestinal microflora (*n* = 3). (**H**) NMDS ordination of bacterial communities (**I**) UPGMA clustering tree based on Unweighted Unifrac distance. Abbreviations: OUT, Operational Taxonomic Unit; PCoA, Principal Coordinate Analysis; NMDS, Non-Metric Multidimensional Scaling; UPGMA, Unweighted Pair Group Method with Arithmetic Mean.

To further investigate the effects of ALP on LPS-induced pulmonary inflammation, ELISA kits were used to measure the levels of inflammatory mediators in BALF (bronchoalveolar. To investigate the effect of LRP on gut microbes in ALI, we analysed the gut flora in the cecum contents of the control, model, and H-LRP groups (referred to as the LRP group) using 16S rRNA sequencing. As shown in Figure 5A, we initially counted sample sparsity curves. The gradual smoothing of these curves indicates sufficient sequencing data. This also indirectly accounts for differences in species abundance across groups. Changes in gut flora abundance between groups were then illustrated by analysing Wayne plots. In the control, model, and LRP groups, we identified 2280, 2223, and 2289 OTUs, including 455, 122, and 151 unique OTUs, and 1471 shared OTUs, respectively. Using the Chao1, Pielou-J, Shannon, and Simpson indices to assess the abundance and diversity of the gut microbiota (Figure 5C–F), we found that these indices decreased in the model group. LRP treatment subsequently restored these indices, indicating an increase in the abundance and diversity of the gut microbiota. In addition, results based on Bray–Curtis Principal Coordinate Analysis (PCoA) and Non-Metric Multidimensional Scaling (NMDS) indicated that the community composition of the model group exhibited greater dispersion compared to the control group. In contrast, the LRP treatment enhanced the similarity of community composition and cohesion with the control group aggregation (Figure 5G,H). The UPGMA hierarchical clustering tree yielded similar results (Figure 5I).

**Figure 6:**
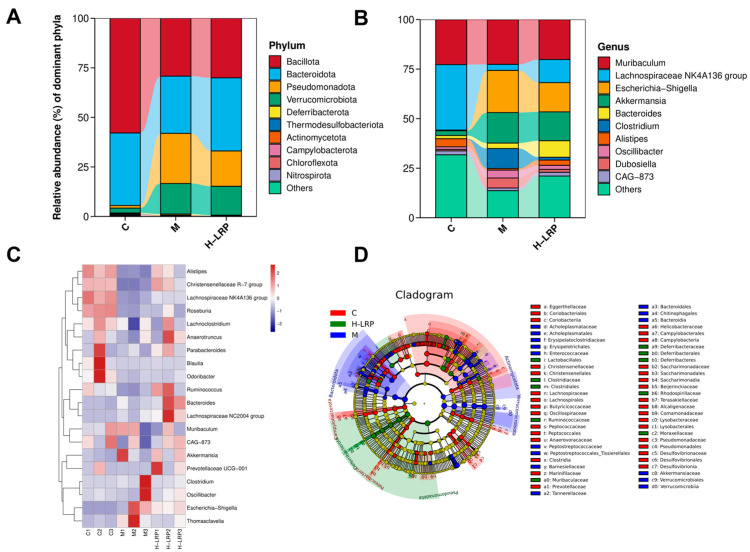
Comparison of the impacts of LRP on gut flora. (**A**,**B**) Relative abundance of intestinal microflora at the phylum and genus levels. (**C**) The heat map depicts the relative abundance of 20 species significantly enriched at the genus level for the three groups of samples (*n* = 3). (**D**) The circos plot shows the correlation between the three groups of samples at the genus level (*n* = 3).

Next, to further explore changes in colony structure, we conducted comparative analyses of the relative abundance of key species at the phylum and genus level, with a particular focus on taxa affected by the LRP. At the phylum level, Firmicutes (Bacillota), Bacteroidetes (Bacteroidota), and Proteobacteria (Pseudomonadota) are the predominant bacterial phyla in the cecal microbiota. Compared to the control group, the Model group exhibited decreased abundances of Bacillota and Bacteroidota, while the abundance of Pseudomonadota increased. LRP treatment increased the abundances of Bacillota and Bacteroidota, and reduced Pseudomonadota abundance (Figure 6A).

At the genus level, the administration of LPS resulted in a reduction in the relative abundance of Muribaculum, while concurrently increasing the abundance of Escherichia-Shigella, Akkermansia, and Clostridium. Treatment with LRP increased the abundance of beneficial bacteria, such as Bacteroides, and reduced the abundance of harmful bacteria, such as Eisenbergiella, in the cecum (Figure 6C). The heat map showed the differences between the intestinal microflora communities of the three groups of mice at the genus.

## 4. Discussion

This study isolated LRP from *Lycium ruthenicum* and demonstrated its antagonistic effects against ALI through triple therapeutic mechanisms. Mice typically develop structural lung damage, inflammatory cell infiltration, and pulmonary edema after LPS exposure [34,35,36]. Pretreatment with LRP significantly attenuated LPS-induced lung injury in mice, characterized by reduced alveolar septal thickening, epithelial cell desquamation, and alveolar collapse, along with ameliorated pulmonary edema. Furthermore, both in vivo and in vitro experiments confirmed that LRP pretreatment effectively suppressed LPS-induced inflammatory responses and mitigated pulmonary inflammatory cell infiltration in ALI mice. Notably, in vitro models suggest that direct LRP action on airway epithelia may involve pattern recognition receptors (e.g., TLR4/Dectin-1): sulfated polysaccharide moieties bind TLR4/MD2 via electrostatic interactions to activate downstream signaling, while β-glucan domains engage Dectin-1 receptors to trigger Rac1/Cdc42 pathway-mediated epithelial repair [37,38]. Critically, it was revealed for the first time that LRP conferred protection via the gut-lung axis, distinguishing it from single-organ targeting strategies. Administration of LRP increased the abundance of potentially beneficial bacteria (Firmicutes/Bacteroidetes) and decreased harmful bacterial populations, thereby restoring gut microbiota homeostasis. Notably, gut-lung microbial crosstalk is increasingly implicated beyond ALI in chronic respiratory diseases like asthma and pulmonary fibrosis, where gut-derived metabolites similarly modulate lung immunity [39].

Inflammation represents a hallmark feature of ALI [40]. As a pivotal pro-inflammatory mediator, TNF-α stimulates the release of other pro-inflammatory cytokines (e.g., IL-1β, IL-6) [41,42] and chemokines (e.g., CXCL8) [43]. Conversely, IL-10 counteracts pro-inflammatory immune responses and protects host tissues from cytokine-mediated damage [44]. Treatment with LRP dose-dependently inhibited neutrophil infiltration in BALF, concurrently downregulating pro-inflammatory cytokines (TNF-α/IL-6) and upregulating the anti-inflammatory cytokine (IL-10). This pleiotropic effect may involve modulation of the NF-κB/MAPK pathway [45], which drives ALI progression, indicating broader mechanistic actions beyond single-pathway inhibitors such as dexamethasone.

Previous studies have established that ALI induces gut dysbiosis and intestinal mucosal barrier disruption [46], while modulation of gut microbiota structure and composition represents a potential therapeutic strategy for ALI [47]. Orally administered LRP does not directly target the airways but acts as a prebiotic substrate for gut commensals. Its β-1,3/1,6-glycosidic bonds are cleaved by glycosidases from specific bacteria (e.g., Bacteroides), generating SCFAs, predominantly acetate, propionate, and butyrate [39]. These SCFAs enter systemic circulation via the portal vein and reach lung tissues. In airways, butyrate suppresses the NLRP3 inflammasome by activating GPR109A receptors on alveolar macrophages, while propionate enhances tight junction protein expression via GPR41 signaling in bronchial epithelium [48]—this “microbial metabolite–distal organ receptor” cascade constitutes the core pathway for LRP-mediated enterogenic pulmonary protection. Molecular evidence suggests that SCFAs from LRP fermentation regulate pulmonary immunity via receptor binding (e.g., GPR41/43) and epigenetic modifications (e.g., HDAC inhibition), though precise molecular targets and spatiotemporal dynamics of polysaccharide-derived metabolites in lung tissue remain uncharacterized [49,50].

Given its synergistic anti-inflammatory and microbiota-modulating effects, LRP may serve as an adjunctive therapy to corticosteroids (e.g., dexamethasone) to enhance lung protection.

Although LRP demonstrated protective potential against ALI in LPS-induced murine models, certain limitations were acknowledged. First, despite identifying SCFAs’ central role, the specific molecular targets of polysaccharide degradation products in the lung remain elusive. Second, the A549 monolayer model fails to recapitulate alveolar 3D architecture and microenvironment, necessitating validation in complex systems like ALI cultures or lung organoids [51]. Third, exclusive use of male mice overlooks sex-dependent immune-microbiome interactions, while the LPS-induced acute injury model inadequately mirrors multifactorial clinical pathologies. Furthermore, the absence of higher mammalian validation and combination therapy trials limits translational potential.

Subsequent studies should integrate isotope tracing to map LRP-SCFAs metabolic flux and identify their dynamic targets in pulmonary immune cells; develop advanced lung models incorporating immune-microbe crosstalk; validate efficacy in female/aged mice and non-human primates under clinically relevant injuries; and prioritize combinatorial strategies with standard therapies to accelerate clinical translation.

## 5. Conclusions

In summary, the anti-inflammatory properties of LRP have been validated in both in vivo and in vitro experiments. In LPS-induced ALI, LRP significantly ameliorated pulmonary tissue morphology, balanced pro- and anti-inflammatory cytokine levels, and restored dysregulated gut microbiota. Our findings suggest that LRP, with its anti-inflammatory and gut microbiota-modulating capabilities, may serve as a potential natural therapeutic agent for lung injury.

## Data Availability

All the data are available from the corresponding author upon reasonable request.

## Abbreviations

The following abbreviations are used in this manuscript:

LRP	*Lycium ruthenicum* Murr. polysaccharide
ALI	Acute lung injury
LPS	Lipopolysaccharide
BALF	Bronchoalveolar lavage fluid
IL-6	Interleukin-6
IL-10	Interleukin-10
IL-1β	Interleukin-1β
TNF-α	Tumor necrosis factor-α
CCK-8	Cell Counting Kit-8
H&E	Staining hematoxylin-eosin staining
PBS	Phosphate-buffer saline
W/D	Wet/Dry Weight Ratio
ELISA	Enzyme-Linked Immunosorbent Assay
OTU	Operational Taxonomic Unit
PCoA	Principal Coordinate Analysis
NMDS	Non-Metric Multidimensional Scaling
UPGMA	Unweighted Pair Group Method with Arithmetic Mean
ANOVA	Analysis of Variance
SD	Standard Deviation
FT-IR	Fourier Transform Infrared Spectroscopy
HPIC	High-Pressure Ion Chromatography
16S rRNA	16S Ribosomal RNA
